# Multichannel Photoluminescence of Graphene Quantum Dots Across Femtosecond to Cryogenic Timescales

**DOI:** 10.1002/smll.202514669

**Published:** 2026-03-05

**Authors:** Hanna Song, Ha Young Lee, Seungkwon Jeon, Seungmin Jeong, Jong Bae Park, Minju Kim, Kwangseuk Kyhm, Robert. A. Taylor, Heedae Kim

**Affiliations:** ^1^ Department of Semiconductor Science & Technology Jeonbuk National University Jeonju Jeonbuk Republic of Korea; ^2^ Research Institute for Materials and Energy Sciences Jeonbuk National University Jeonju Jeonbuk Republic of Korea; ^3^ Korea Basic Science Institute Jeonju Centre Jeonju Jeonbuk Republic of Korea; ^4^ Department of Opto & Cogno Mechatronics Engineering Pusan National University Busan Republic of Korea; ^5^ Department of Physics University of Oxford Oxford UK

**Keywords:** cryogenic photoluminescence, femtosecond dynamics, graphene quantum dots, optical kerr gating, time‐correlated single photon counting

## Abstract

Graphene quantum dots (GQDs) exhibit complex photoluminescence (PL) originating from intrinsic sp^2^ carbon domains, surface functional groups, and structural defects. Yet the spectral overlap among these emissive channels hinders clear identification of their recombination pathways. Here, we investigate multichannel PL dynamics of commercial GQDs using time‐resolved and cryogenic PL spectroscopy. PL spectra reveal three distinct peaks: Peak I (443 nm) from π–π* transitions, Peak II (520 nm) from surface‐dominated contribution functional states, and Peak III (583 nm) from pyrrolic N‐related defects. Time‐correlated single‐photon counting detects only a 460 nm emission linked to graphitic N traps, indicating that Peaks I–III decay faster than the nanosecond window. Ultrafast optical Kerr‐gate measurements further resolve distinct lifetimes for hydroxyl (<5 ps), carboxyl (5–10 ps), amine (20–30 ps), and carbonyl (40–80 ps) groups. The transient evolution displays cascade relaxation from deep to shallow traps, evidenced by a progressive blue‐shift of Peak II. Cryogenic PL shows stable emission of Peak I, whereas Peak III red‐shifts and broadens with temperature, revealing strong electron–phonon coupling and deep‐level trapping. These results clarify the multichannel emission mechanisms of GQDs and provide design principles for tuning their optical properties.

## Graphical abstract

This study reveals how graphene quantum dots emit light across all relevant timescales, from femtoseconds to cryogenic steady states. By integrating optical Kerr gating, time‐correlated single‐photon counting, and low‐temperature photoluminescence, distinct intrinsic, surface, and defect‐related emission channels are temporally and spectrally disentangled, providing a unified multichannel photoluminescence dynamics.

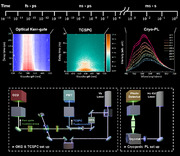

## 1 Introduction

Graphene quantum dots (GQDs), a novel class of zero‐dimensional carbon nanomaterials, have garnered increasing attention due to their outstanding mechanical properties [1], biocompatibility [2], and facile structural tunability [3]. Unlike bulk graphene, GQDs exhibit unique (e.g., size‐ and edge‐dependent) photoluminescence (PL), opening avenues for applications in bioimaging [4], optical sensing [5], photocatalytic [6], and next‐generation optoelectronic devices [7]. Despite extensive studies, the origin of their emission remains elusive, as it stems from a complex interplay among intrinsic π–π* transitions [8], surface functional states [9], and defect‐related states [10].

While numerous efforts have been made to elucidate the PL mechanisms of GQDs by varying excitation wavelengths [11], solution pH [12], particle size [13], concentration [14], and dispersion media [15], these approaches have provided only limited insight into the fundamental electronic processes governing GQD emission. To enhance the typically weak intrinsic emission of GQDs, post‐synthetic treatments such as nitrogen (N) doping [16] and surface functionalization [17] have been widely employed, which can indeed improve PL intensity [18] and stability [19]. However, these modifications also introduce various emissive states, further complicating the interpretation of GQD photo‐physics. Critically, each emissive component exhibits distinct recombination dynamics, rendering it fundamentally challenging to resolve all emission pathways using a single spectroscopic technique.

In this paper, we systematically examine the multichannel emission mechanisms of commercial GQDs by integrating time‐resolved spectroscopy and cryogenic PL analysis. The time‐resolved approach combines time‐correlated single‐photon counting (TCSPC) and optical Kerr‐gate (OKG) spectroscopy within a unified experimental platform, enabling access to photoluminescence dynamics over a broad temporal range. TCSPC probes nanosecond‐scale radiative recombination by recording photon arrival times, selectively highlighting emissive channels with longer lifetimes. In contrast, OKG spectroscopy directly captures ultrafast emission dynamics on femtosecond‐to‐picosecond timescales through optical gating. The combination of TCSPC and OKG therefore enables complementary access to slow and ultrafast photoluminescence processes, providing a comprehensive view of multichannel emission dynamics beyond the reach of a single time‐resolved technique. Room‐temperature PL spectra reveal multiple emissive pathways, which are structurally correlated via x‐ray photoelectron spectroscopy (XPS), Fourier‐transform infrared spectroscopy (FTIR), and UV–vis absorption analysis. To probe their temporal dynamics, we employ TCSPC and OKG spectroscopy, each offering distinct temporal resolutions. Notably, the ultrafast sensitivity of OKG enables the detection of activation behavior linked to specific surface functional groups, which are inaccessible to nanosecond‐resolved TCSPC. Furthermore, cryogenic PL spectroscopy reveals otherwise obscured intrinsic emissions and defect‐state contributions—particularly those associated with pyrrolic N—which are unresolved in time‐domain analyses. This comprehensive spectroscopic approach disentangles the contributions of intrinsic transitions, surface states, and defect‐related traps, offering a unified understanding of GQD emission.

## 2 Results and Discussion

### 2.1 Schematic Diagram of Functionalized GQDs

**FIGURE 1:**
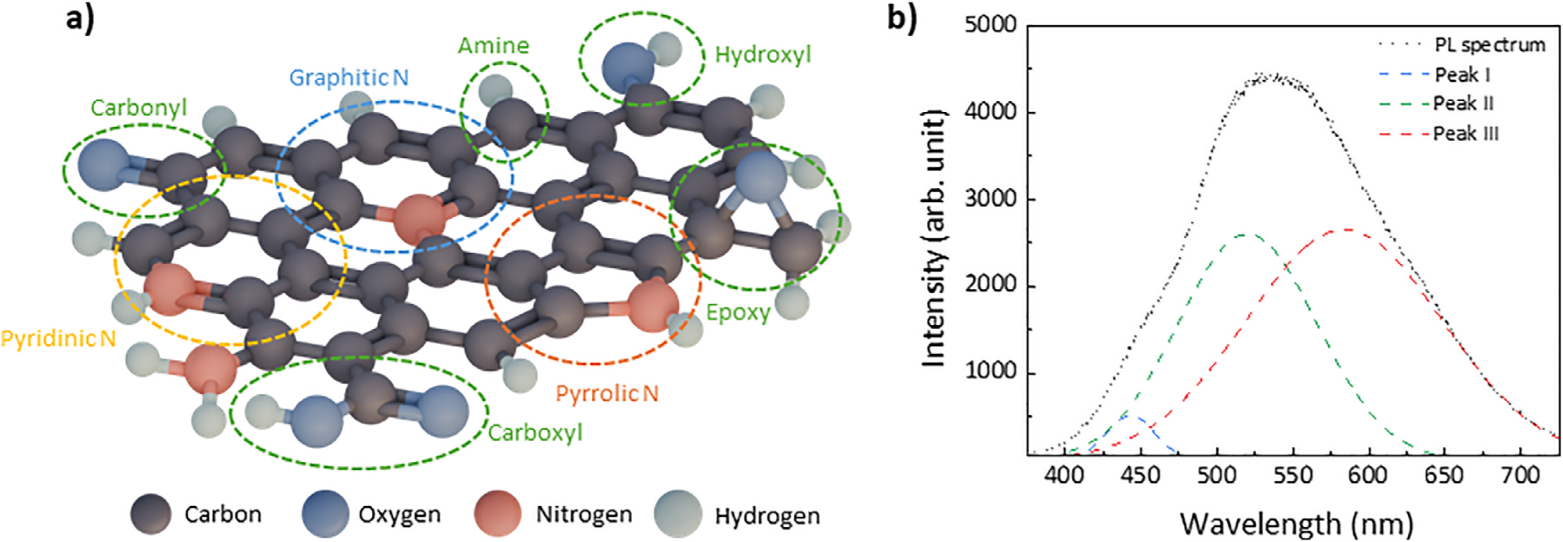
Structural and optical analysis of functionalized graphene quantum dots (GQDs). (a) Conceptual atomic illustration of a graphene quantum dot (GQD) highlighting representative dopants and functional groups, including graphitic N, pyridinic N, pyrrolic N, hydroxyl, carbonyl, carboxyl, and amine functional groups. (b) Room‐temperature photoluminescence (PL) spectrum of GQDs under 325 nm excitation, showing a broad asymmetric emission band. The experimental spectrum (dotted line) is deconvoluted into three components corresponding to Peak I (443 nm), Peak II (520 nm), and Peak III (583 nm) (dashed lines). Data are representative of at least three independent measurements (n ≥ 3). Dashed lines indicate Gaussian fitting.

To investigate the origin of the multiband emission in GQDs, a commercial aqueous GQD solution (0.005 wt.%, Sigma–Aldrich) was drop‐cast onto silicon substrates and characterized (Figure S1). The detailed structural characteristics of the GQDs were characterized by transmission electron microscopy (TEM), as shown in the Figure S2. Figure 1a presents a schematic and representative structural model of a GQD, constructed based on spectroscopic analysis rather than a direct atomic‐scale reconstruction, highlighting graphitic, pyridinic, and pyrrolic N dopants along with various oxygen‐containing functional groups. In GQDs, intrinsic photoluminescence arises primarily from π–π* transitions within sp^2^‐hybridized carbon domains [20]. However, the presence of heteroatom dopants (e.g., nitrogen) and functional moieties such as hydroxyl, carboxyl, and carbonyl groups introduces localized energy states within the π‐conjugated system [21]. These states act as trap sites or intermediate levels, facilitating diverse radiative recombination pathways beyond the intrinsic transition [22]. Figure 1b shows the room‐temperature PL spectrum of GQDs measured under 325 nm excitation, featuring a broad and asymmetric emission band. Gaussian deconvolution resolves this into three distinct peaks: Peak I (443 nm, high‐energy), Peak II (520 nm, intermediate), and Peak III (583 nm, low‐energy). The adoption of a three‐peak model is supported not only by fitting quality and the reproducibility of peak positions across temperature‐ and power‐dependent measurements, but also by their consistency with chemical states independently identified by complementary spectroscopic analyses, as discussed in detail below. This spectral structure suggests the coexistence of multiple emissive centers, including intrinsic sp^2^ carbon core transitions, surface‐related states, and defect‐induced emissions. These results indicate that electron–hole recombination in GQDs occurs via multiple emission pathways, reflecting the heterogeneous nature of their structural and electronic environments [23].

### 2.2 Chemical Characterization of GQDs

**FIGURE 2:**
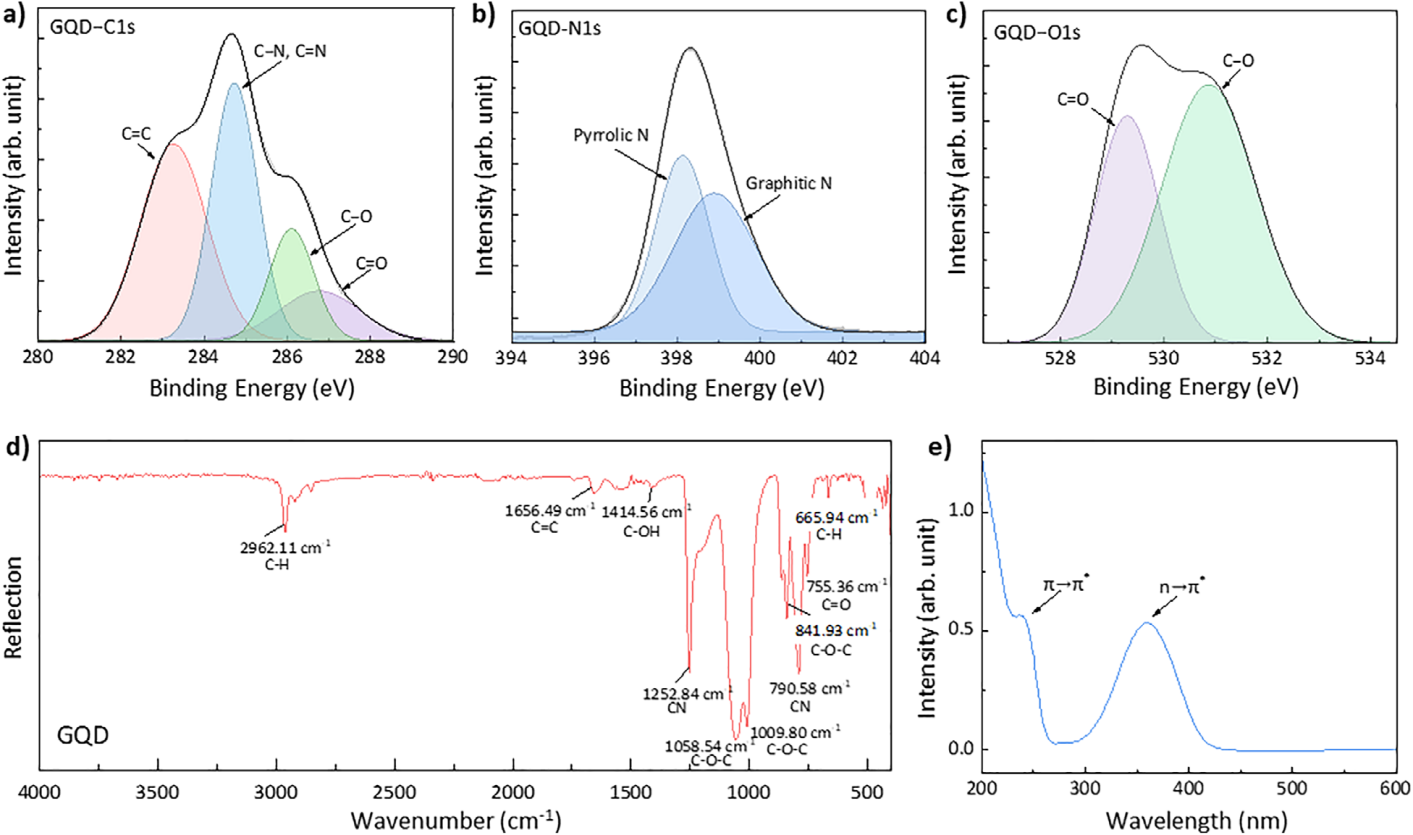
Spectroscopic identification of chemical and defect‐related emission centers in GQDs. (a) XPS C1s spectrum showing contributions from C═C, C─N/C═N, C─O, and C═O bonds. (b) N1s spectrum displaying graphitic N and pyrrolic N, linked to shallow and deep trap states, respectively. (c) O1s spectrum with peaks from C═O and C─O bonds. (d) FTIR spectrum indicating the presence of sp^2^ carbon and various oxygen functional groups. (e) UV–vis absorption spectrum showing π–π* and n–π* transitions. All spectra were acquired from at least three independent measurements (*n* ≥ 3).

To further understand the origin of the multiband emissions observed in the room‐temperature PL spectrum (Figure 1b), the chemical composition and electronic structure of GQDs were analyzed using XPS, FTIR spectroscopy, and UV–vis absorption spectroscopy. The C 1s spectrum (Figure 2a) reveals signals at 283.2 eV (C═C), 284.7 eV (C─N/C═N), 286.1 eV (C─O), and 286.8 eV (C═O), indicating a sp^2^ carbon network decorated with nitrogen and oxygen functional groups [24]. The prominent C═C peak supports the attribution of Peak I (443 nm) to intrinsic π–π* transitions within the conjugated carbon domains. The N 1s spectrum (Figure 2b) shows contributions from graphitic N (∼398.9 eV) and pyrrolic N (∼398.1 eV) [24]. Graphitic N, incorporated substitutionally into the carbon lattice, introduces shallow donor‐like states with minimal perturbation of the π‐system, consistent with weak modulation of intrinsic emission energies [25, 26]. In contrast, pyrrolic N, embedded in five‐membered rings, induces deep trap states within the bandgap and contributes to the red‐shifted Peak III (583 nm) through orbital hybridization effects [27, 28]. The O 1s spectrum (Figure 2c) further confirms the presence of C═O (529.2 eV) and C─O (530.8 eV) bonds [29]. FTIR analysis (Figure 2d) identifies vibrational modes from C═C, C─OH, C─O─C, and C═O groups, validating the presence of various surface oxygen functionalities. These species serve as trap sites facilitating thermally activated radiative recombination, aligning with the intermediate‐energy Peak II (520 nm) [30, 31]. The UV–vis absorption spectrum (Figure 2e) displays features at ∼230 nm (π–π* transitions from C═C) and ∼360 nm (n–π* transitions from C═O/C─O), corroborating the assignment of Peak II to oxygen‐related functional states that are predominantly localized at or near the surface of the GQDs [22, 32, 33].

### 2.3 Time‐Resolved PL and OKG Measurements

**FIGURE 3:**
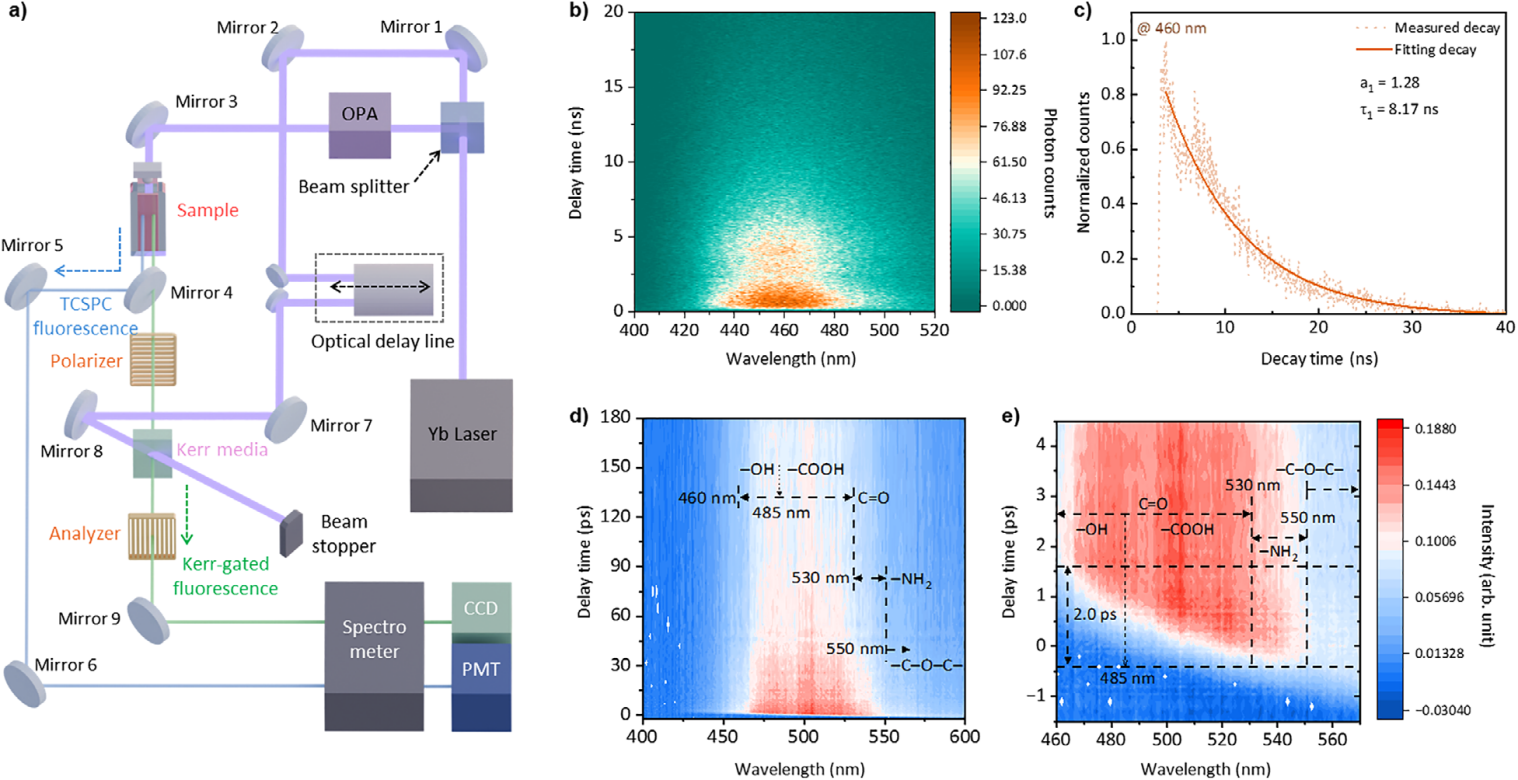
Time‐resolved photoluminescence and ultrafast Kerr gate analysis of GQD emissive pathways. (a) Schematic of the integrated setup for time‐correlated single‐photon counting (TCSPC) and optical Kerr gate (OKG) measurements. The emission pathway is switched by adjusting Mirror 4, allowing seamless transition between TCSPC (detected via photomultiplier tube; PMT) and OKG (detected via charge‐coupled device; CCD) modes within a unified optical configuration. (b) TCSPC map displaying the spectral–temporal emission distribution over the 400–520 nm range, with a dominant emission centered at 460 nm. (c) Corresponding decay profile at 460 nm fitted with a mono‐exponential function (τ ≈ 8.17 ns). (d) Time–wavelength‐resolved fluorescence map acquired via the optical Kerr gate (OKG) technique, revealing strong sub‐picosecond emission within the Peak II region. (e) Magnified view of early delay region (─1.5 to 4.5 ps), showing a spectral blue‐shift at the 460–550 nm, indicative of sequential recombination from functional trap states (e.g., amide, carbonyl, carboxyl, and hydroxyl). Data were obtained from at least three independent measurements (*n* ≥ 3). Decay curves were analyzed using mono‐ or bi‐exponential fitting.

While steady‐state photoluminescence reflects the time‐integrated emission dominated by channels with high radiative yield, TCSPC selectively probes emissive pathways that survive within the nanosecond temporal window, providing complementary insight into recombination dynamics that are not necessarily reflected in steady‐state spectra. Ultrafast relaxation on femtosecond‐to‐sub‐picosecond timescales is widely reported in sp^2^ carbon systems due to efficient carrier–carrier and carrier–phonon scattering, and ultrafast broadband emission has been observed under femtosecond excitation in graphene [34]. Consistently, femtosecond transient absorption studies on graphene quantum dots also reveal ultrafast excited‐state dynamics, supporting that an intrinsic sp^2^‐domain–related channel may contribute within the OKG‐accessible time window [35]. To investigate the temporal characteristics of multiple emission channels in GQDs, we performed TCSPC and optical Kerr‐gate (OKG) spectroscopy. Figure 3a illustrates the integrated optical setup that enables both measurements within a shared configuration. The GQD sample was measured in solution form using a standard quartz cuvette (Figure S3). By adjusting a single mirror (Mirror 4), the emitted signal was directed either to a photomultiplier tube (PMT) for TCSPC or to a charge‐coupled device (CCD) for OKG detection, allowing efficient acquisition of nanosecond‐ and sub‐picosecond‐resolved fluorescence data. Detailed optical layouts and component specifications are provided in Figure S4 and Tables S1–S2. As shown in Figure 3b, the TCSPC map displays the spectral–temporal emission distribution over the 400–520 nm range, with a dominant signal centered around 460 nm. The corresponding decay profile (Figure 3c) exhibits mono‐exponential behavior with a lifetime of approximately 8.17 ns. This emission is tentatively attributed to trap states associated with graphitic N dopants [36], consistent with the XPS analysis presented in Figure 2. Notably, none of the three‐characteristic steady‐state PL peaks at 443, 520, and 583 nm are clearly resolved in the TCSPC map. The emission centered at ∼460 nm observed in TCSPC does not directly correspond to the steady‐state PL peaks but instead represents a temporally selective recombination pathway that persists within the nanosecond detection window. Due to its relatively low radiative efficiency compared to ultrafast emission channels, this contribution is weak in steady‐state PL spectra but becomes pronounced in time‐resolved measurements. Temperature‐dependent TCSPC measurements under cryogenic conditions further reveal a monotonic increase in lifetime with decreasing temperature, consistent with the suppression of non‐radiative recombination pathways typically observed for shallow emissive states in graphene‐based quantum dots (Figure S5) [37]. Based on these observations, we infer that Peaks I and II undergo ultrafast decay beyond the temporal resolution of TCSPC. In contrast, the absence of Peak III in time‐resolved measurements is attributed primarily to its low radiative efficiency and dominant non‐radiative recombination pathways, rather than to an ultraslow radiative decay, rendering it effectively undetectable via photon counting.

To further probe the ultrafast regime, OKG measurements were performed. Potential artifacts associated with optical Kerr‐gate measurements, including the intrinsic Kerr medium response, cross‐phase modulation, and Raman scattering, were carefully considered. The OKG signal was recorded within a well‐defined temporal window and spectrally filtered to suppress scattered excitation and Raman contributions. Control measurements at negative delay times confirmed the absence of spurious background signals. As shown in Figure 3d, the time–wavelength‐resolved fluorescence map reveals a strong sub‐picosecond signal within the Peak II spectral window (460–560 nm), indicating the presence of temporally distinct emission pathways inaccessible via TCSPC. To quantitatively resolve these pathways, we performed wavelength‐dependent lifetime fitting based on prior literature assignments of functional groups to specific spectral regions. When fitting was performed separately for each functional group–dominant band, distinct lifetime components were observed. The amine (–NH_2_)‐related band (530–550 nm) [38] exhibited a single decay component of 20–30 ps (Figure S6). The carboxyl (–COOH)‐dominant band (485–530 nm) [38], which overlaps with carbonyl (C═O) contributions [39], showed dual decay components of 5–10 ps and 40–80 ps (Figure S7). Similarly, the hydroxyl (─OH)‐related band (460–485 nm) [22], also overlapping with carbonyl (C═O) emission, exhibited dual lifetimes of <5 ps and 40–80 ps (Figure S8). Notably, since both hydroxyl and carboxyl groups form shallow traps stabilized by hydrogen bonding and display sub‐10 ps fast decay components, distinguishing their contributions based solely on lifetime is challenging. However, it is generally recognized that hydroxyl‐related emission arises at shorter wavelengths compared to carboxyl‐related emission. Accordingly, the observed spectral evolution suggests a natural blue‐shift from carboxyl‐dominant to hydroxyl‐dominant regions. In addition, the presence of a relatively long‐lived component (∼40–80 ps) in both spectral regions is attributed to deeper trap states associated with carbonyl (C═O) groups. In contrast, amine groups act as donor‐type traps, yielding a relatively longer lifetime (∼30 ps). Carbonyl groups, associated with n–π* transitions, exhibited the longest lifetimes (∼40–80 ps), consistent with their relatively deep trap nature. Notably, epoxy (C─O─C) groups are known to suppress radiative emission through dominant non‐radiative pathways, and their contribution in the >550 nm region was correspondingly weak [22], with only minor emission signals observed and a measured single decay component of ∼40 ps (Figure S9). Thus, epoxy‐related emission remained weak and did not significantly contribute to the observed cascade dynamics. The overall temporal evolution (Figure 3e) reflects this cascade relaxation mechanism: amine‐related emission (530–550 nm) appear within ∼0.2 ps, followed by carboxyl/carbonyl‐related emissions (485–530 nm) emerging during ∼0.7 ps, and finally hydroxyl‐related signals (460–485 nm) becoming prominent until ∼2.0 ps. This sequential relaxation behavior supports a model in which carriers first localize in deeper trap states and subsequently transfer to shallower traps before radiative decay. After ∼2.0 ps, no further spectral evolution is observed, indicating that the major surface‐state recombination processes are complete.

In prior time‐resolved measurements using TCSPC and OKG techniques, neither Peak I nor Peak III emissions were distinctly resolved. Instead, a distinct emission centered at 460 nm was observed and tentatively attributed to trap states introduced by graphitic N embedded within the sp^2^ carbon framework (see Figure 3). This result suggests that Peak I and Peak III either undergo ultrafast non‐radiative recombination or emit outside the temporal detection window of both techniques.

### 2.4 Cryogenic PL characteristics of GQDs

**FIGURE 4:**
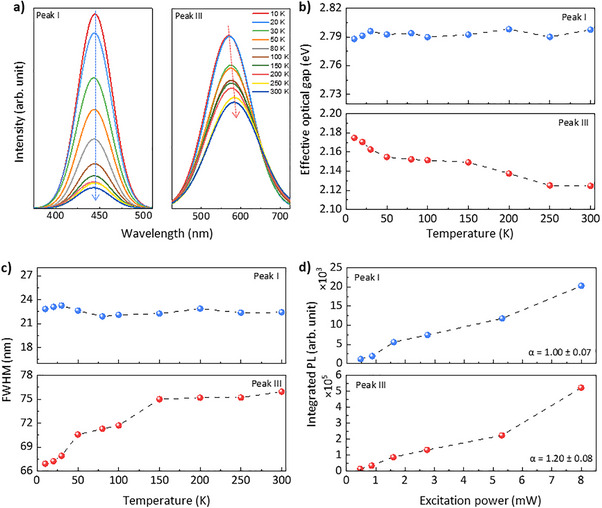
Various PL characteristics and defect‐state emission analysis in GQDs. (a) Extracted PL spectra of Peak I and Peak III measured from 10 to 300 K. As temperature increases, the PL intensity of both peaks decreases. While the spectral position of Peak I remains nearly unchanged, Peak III exhibits a gradual red‐shift. (b) Extracted temperature‐dependence effective optical gap of Peak I and Peak III as a function of temperature. The energy bandgap values were extracted from the center energies of the Gaussian‐fitted PL peaks and represent effective optical gaps associated with radiative recombination. (c) Extracted temperature‐dependence of the full width at half maximum (FWHM) for both emission peaks. (d) Integrated PL intensity of Peak I and Peak III as a function of excitation power. The extracted power factor α is approximately 1.00 for Peak I, indicative of intrinsic excitonic recombination, and 1.20 for Peak III, consistent with defect‐mediated emission mechanisms. Data were obtained from at least three independent measurements at each condition (*n* ≥ 3). Solid lines represent fitting results.

It should be noted that the absence of certain emissive features in time‐resolved measurements does not imply the absence of the corresponding radiative states. Rather, it reflects the temporal selectivity and sensitivity limits of the measurement techniques. Under steady‐state cryogenic conditions, where non‐radiative processes are suppressed, these emissive states can be stably observed and quantitatively analyzed. To clarify the emission mechanism of Peak I and III, temperature‐dependent PL spectroscopy was conducted under cryogenic conditions. Figure 4a presents the temperature‐dependent PL behavior of Peak I and Peak III, extracted from full‐spectrum measurements over the 10–300 K range (see Figure S10 for full spectra). With increasing temperature, the PL intensity of both peaks progressively decreased. Notably, while Peak I maintained a nearly fixed spectral position, Peak III exhibited a progressive red‐shift. This trend suggests the involvement of thermally activated non‐radiative decay channels rather than Auger‐type processes, as Auger recombination rates scale with the cube of the carrier density (n^3^ dependence), leading to ultrafast quenching signatures that are not observed in the present time‐resolved measurements [40, 41]. At all temperatures, the emission spectra could be consistently deconvoluted into three Gaussian components (Figure S11), indicating the presence of distinct emissive states governed by independent recombination pathways. Among these, Peak III showed the most significant spectral red‐shift with increasing temperature (Figure 4b), which can be attributed to thermal expansion and strong electron–phonon coupling [42]. In contrast, Peak I exhibited negligible spectral movement, highlighting a fundamental difference in their electronic origins. This contrast is further supported by the temperature dependence of the full width at half maximum (FWHM), shown in Figure 4c. Peak III exhibited pronounced spectral broadening at elevated temperatures, reflecting increased phonon coupling and impurity scattering. Meanwhile, Peak I showed minimal or even slightly reduced FWHM, reinforcing its assignment as an intrinsic recombination center [43]. The emission band of Peak III (centered near 583 nm) also aligns well with previously reported PL from pyrrolic N defects, typically observed in the 570–620 nm range [28]. These defects are known to possess long‐lived, vibrationally coupled states with high sensitivity to lattice dynamics, consistent with the thermal fragility and slow kinetics inferred here. In addition to thermal behavior, the excitation power dependence of Peak I and Peak III was investigated to probe their recombination dynamics (Figure 4d). The full power‐dependent PL spectra are presented in the Figure S12. Each spectrum was deconvoluted into three Gaussian components following the same fitting procedure used in Figure 4a (see Figure S13), allowing the individual contributions of Peak I and Peak III to be quantitatively extracted. The integrated intensities of the two peaks were then plotted as a function of excitation power and fitted using a power‐law relation of the form *I ∝ P^α^*, where *I* denotes the integrated peak intensity and *P* is the incident laser power [44]. The extracted power factor *α* was approximately 1.0 for Peak I and 1.2 for Peak III. The linear behavior of Peak I is characteristic of band‐edge excitonic emission, whereas the super‐linear response of Peak III further supports its origin in carrier trapping and defect‐mediated recombination, consistent with its assignment to localized defect states. These distinct power‐dependent characteristics provide further evidence that Peak III originates from localized defect states, while Peak I arises from intrinsic π–π* transitions within the sp^2^ carbon framework.

## 3 Conclusion

In summary, we systematically elucidated the multichannel photoluminescence mechanisms of GQDs by integrating time‐resolved and cryogenic PL spectroscopy. Room‐temperature PL spectra revealed three distinct emission bands, corresponding to intrinsic sp^2^‐domain transitions (Peak I), functionalized surface states (Peak II), and pyrrolic N‐related defects (Peak III). Time‐resolved measurements uncovered distinct temporal characteristics for each emissive channel. TCSPC detected only a 460 nm emission associated with graphitic N traps, indicating that Peaks I, II, and III either decay non‐radiatively or lie outside the nanosecond window. In contrast, sub‐picosecond resolution OKG measurements quantitatively resolved ultrafast emission dynamics from Peak II, revealing distinct lifetime components attributed to specific surface functional groups: hydroxyl‐related bands exhibited <5 ps and 40–80 ps dual lifetimes, carboxyl/carbonyl bands displayed 5–10 ps and 40–80 ps lifetimes, and amine‐related bands showed single lifetimes of 20–30 ps. This spectral and temporal evolution, consistent with a cascade relaxation model, underscores the heterogeneous recombination pathways arising from surface functionalization. Cryogenic PL analysis further differentiated thermal behavior: Peak I remained spectrally stable, whereas Peak III exhibited pronounced red‐shift and spectral broadening with increasing temperature, indicating strong electron–phonon coupling and deep‐level trapping. Power‐dependent PL analysis revealed a linear power factor (α ≈ 1.0) for Peak I and a super‐linear factor (α ≈ 1.2) for Peak III, reinforcing their respective intrinsic and defect‐mediated origins. Collectively, these findings provide a unified understanding of the temporally and spectrally distinct emission pathways in GQDs, offering valuable insights for the rational tuning of their optical properties via controlled doping and surface engineering for applications in optoelectronics, sensing, and bioimaging.

## 4 Experimental Section/Methods

### 4.1 Time‐Resolved Photoluminescence Spectroscopy

Time‐resolved photoluminescence measurements were carried out using a time‐correlated single‐photon counting system (HARPIA‐TF, Light Conversion Ltd.) equipped with a pulsed diode laser (λ = 325 nm, pulse width <204 fs, repetition rate = 200 kHz) as the excitation source at Jeonbuk National University. The emitted signal was spectrally dispersed by a monochromator and detected using a hybrid photomultiplier tube (HPM). The instrument response function (IRF) of the TCSPC system was measured using a 100‐fs fiber laser under identical detection conditions and exhibited a full width at half maximum of approximately 20 ps, which is significantly shorter than the extracted nanosecond‐scale decay times. The IRF is provided in Figure S14.

Ultrafast fluorescence dynamics were investigated using an OKG setup with a YAG crystal femtosecond laser (CB3‐40 W, Light Conversion Ltd.) as the light source. The pump beam was frequency‐doubled to 325 nm for sample excitation, while the gate beam was used to modulate a Kerr medium (SF57) placed between two crossed polarizers. The temporal resolution of the system was approximately ≥400 fs (full‐width at half‐maximum), enabling selective detection of ultrafast fluorescence dynamics in the picosecond regime.

### 4.2 Integrated TCSPC‐OKG Experimental Setup

The integrated TCSPC–OKG experimental setup employed in this work was designed to share a common imaging spectrograph, enabling seamless switching between nanosecond‐ and ultrafast‐resolved photoluminescence measurements within a unified optical configuration. Both TCSPC and OKG measurements were performed using the same imaging spectrograph (Kymera 193i, Andor Technology Ltd), while the detection mode was selected by exchanging the detector and redirecting the emission path using magnetic mirrors.

For TCSPC measurements, the spectrally dispersed emission was detected using a photomultiplier tube (PMT, HPM‐100‐07, Becker & Hickl GmbH) and processed by commercially available TCSPC timing electronics. For OKG measurements, the emission was detected using a charge‐coupled device (CCD, S7031, Hamamatsu Photonics) detector mounted on the same spectrograph. The transition between TCSPC and OKG modes was achieved by changing the detector without altering the core optical alignment, ensuring consistent spectral conditions for both measurements.

In OKG measurements, the time zero was defined as the temporal overlap between the fluorescence signal and the gate pulse, corresponding to the onset of the gated emission signal. Temporal delay was controlled using a mechanical optical delay line, in which displacement of the retroreflector toward the left increased the temporal separation between the pump and gate pulses.

### 4.3 Cryogenic Photoluminescence Spectroscopy

PL measurements at cryogenic temperatures were performed using a closed‐cycle helium cryostat system (E31E‐0E00‐40, Cryo H&I, Inc) equipped with a temperature controller (Model 325, Lake Shore Cryotronics, Inc.), allowing precise control from 10 to 300 K. Both cryogenic and room‐temperature PL measurements were carried out under identical excitation conditions. The sample was excited using a continuous‐wave 325 nm He‐Cd laser (IK3552R‐G, Kimmon Koha Co., Ltd), and the emitted PL was collected in a backscattering geometry and dispersed by a monochromator (SP2500i, Acton) onto a thermoelectrically cooled CCD detector (PIXIS 7515‐0001, Acton). All spectra were corrected for system response.

### 4.4 Cryogenic TCSPC Spectroscopy

Cryogenic TCSPC measurements were performed using a closed‐cycle helium cryostat (CryoAdvance 50, Montana Instruments), allowing temperature control from 4 to 300 K. Samples were excited by an 80 ps pulsed diode laser (LDH‐FA, PicoQuant) operating at 355 nm. The emitted photoluminescence was collected in a backscattering geometry and spectrally selected using a monochromator (Monora 750i, DXG). The signal was detected by a PMT (PHD‐400, Becker & Hickl), and photon arrival times were analyzed using a commercial TCSPC module (SPC‐180N, Becker & Hickl).

### 4.5 Material Characterization

TEM measurements were performed using a transmission electron microscope (Talos F200X G2, ThermoFisher) to characterize the lateral size of the GQDs. XPS measurements were conducted using a Thermo Fisher Scientific K‐Alpha spectrometer equipped with a monochromatic Al Kα x‐ray source. The pass energy was set to 50 eV for high‐resolution scans. FT‐IR spectra were recorded using a Perkin Elmer Spectrum 3 spectrometer equipped with a signal‐to‐noise ratio of 50 000:1 (peak‐to‐peak, 1 min acquisition), covering the range of 350–7800 cm^−1^ with a spectral resolution of 0.4 cm^−1^ installed in the Center for University‐wide Research Facilities (CURF) at Jeonbuk National University. UV–vis absorption spectra were measured using a UV–vis/NIR Spectrometer (V‐780, Jasco) spectrophotometer in the wavelength range of 200–800 nm. The samples were prepared by drop‐casting the GQD solution onto Si substrate (100) and subsequently dried on a hot plate at 100°C.

### 4.6 Statistical Analysis

All spectroscopic data were pre‐processed following standard procedures. Raw photoluminescence spectra were corrected for system response and background signals prior to analysis. Spectral normalization was applied where appropriate to enable comparison of relative intensity distributions. No data points were excluded as outliers unless affected by obvious instrumental artifacts.

Photoluminescence spectra were deconvoluted using Gaussian fitting to extract peak positions, full width at half maximum (FWHM), and integrated intensities. Time‐resolved decay curves obtained from TCSPC and optical Kerr‐gate measurements were fitted using mono‐ or bi‐exponential models to determine characteristic lifetimes. Data are presented as mean values with fitting uncertainties or standard deviations, as appropriate.

For steady‐state and temperature‐dependent measurements, the sample size (n) corresponds to the number of independently acquired spectra at each experimental condition, with *n* ≥ 3 for all reported analyses. Power‐dependent photoluminescence data were analyzed using a power‐law relationship (*I ∝ P^α^*), where the exponent ∝ was extracted by linear regression on a logarithmic scale.

Formal hypothesis testing and statistical significance tests (e.g., *P* values) were not applied, as the analysis focused on parameter extraction and comparative trends rather than group‐wise statistical inference. All data analysis and fitting procedures were performed using OriginPro (OriginLab Corporation).

## Author Contributions

H.Y.L. and H.S. conceived and designed the experiments. H.S. performed the cryogenic PL, UV–vis/NIR Spectrometer, and TEM measurements. S.J. conducted the TCSPC measurements, and S. Jeong carried out the optical Kerr gate experiments. J.B.P. performed the XPS analysis. M.K. and K.K. performed the cryogenic TCSPC measurements. H.Y.L. and H.S. wrote the original draft. R.A.T. and H.K. reviewed and edited the manuscript and supervised the project. All authors discussed the results and contributed to the final version of the manuscript.

## Conflicts of Interest

The authors declare no conflicts of interest.

## Supporting information




**Supporting File**: smll73019‐sup‐0001‐SuppMat.docx.

## Data Availability

The data that support the findings of this study are available from the corresponding author upon reasonable request.
