# Supplementary material for: Multichannel Photoluminescence of Graphene Quantum Dots Across Femtosecond to Cryogenic Timescales
Source: Small. 2026 Mar 5;22(24):e14669. doi: 10.1002/smll.202514669 (PMC13114493; doi:10.1002/smll.202514669)
Supplement: Supplementary file 1 — Supporting File: smll73019‐sup‐0001‐SuppMat.docx. [file SMLL-22-e14669-s001.docx]

Supporting Information

Multichannel Photoluminescence of Graphene Quantum Dots across Femtosecond to Cryogenic Timescales

Hanna Song^†^, Ha Young Lee^†^, Seungkwon Jeon, Seungmin Jeong, Jong Bae Park, Minju Kim, Kwangseuk Kyhm, Robert. A. Taylor* & Heedae Kim*

† These authors contributed equally to this work.

* Corresponding author

Hanna Song, Ha Young Lee, Seungkwon Jeon, Seungmin Jeong, Heedae Kim
Department of Semiconductor Science & Technology, Jeonbuk National University, Jeonju, Jeonbuk 54896, Republic of Korea.
E-mail: hdkim1@jbnu.ac.kr (H. Kim)

Hanna Song, Ha Young Lee, Seungkwon Jeon, Seungmin Jeong, Heedae Kim
Research Institute for Materials and Energy Sciences, Jeonbuk National University, Jeonju, Jeonbuk 54896, Republic of Korea.

Jong Bae Park
Jeonju Centre, Korea Basic Science Institute, Jeonju, Jeonbuk 54896, Republic of Korea.

Minju Kim, Kwangseuk Kyhm

Department of Opto & Cogno Mechatronics Engineering, Pusan National University, Busan 46241, Republic of Korea.

Robert. A. Taylor
Department of Physics, University of Oxford, Parks Road, Oxford OX1 3PU, United Kingdom.
E-mail: Robert.Taylor@physics.ox.ac.uk (R. A. Taylor)


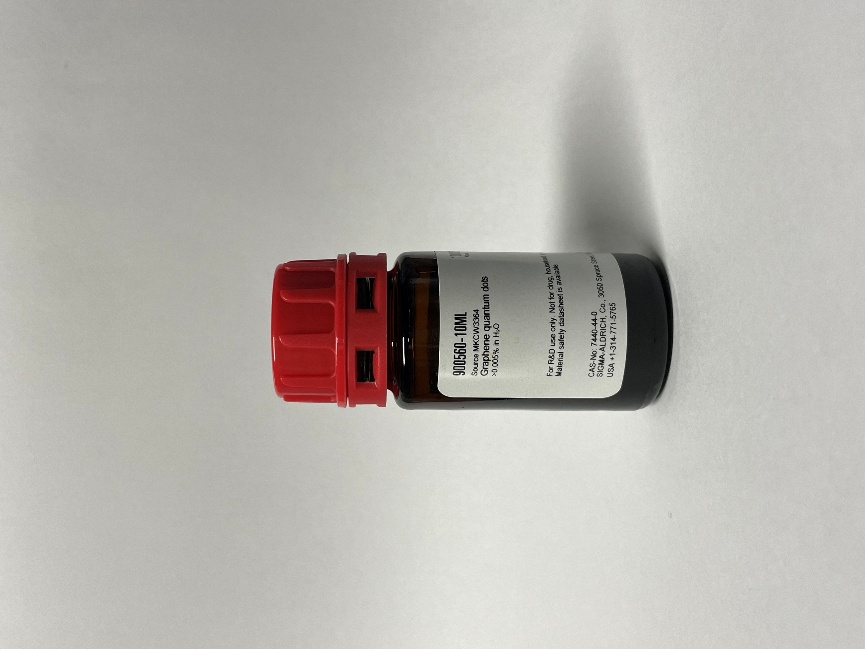


**Figure S1.** Photo image of commercial aqueous GQD solution.
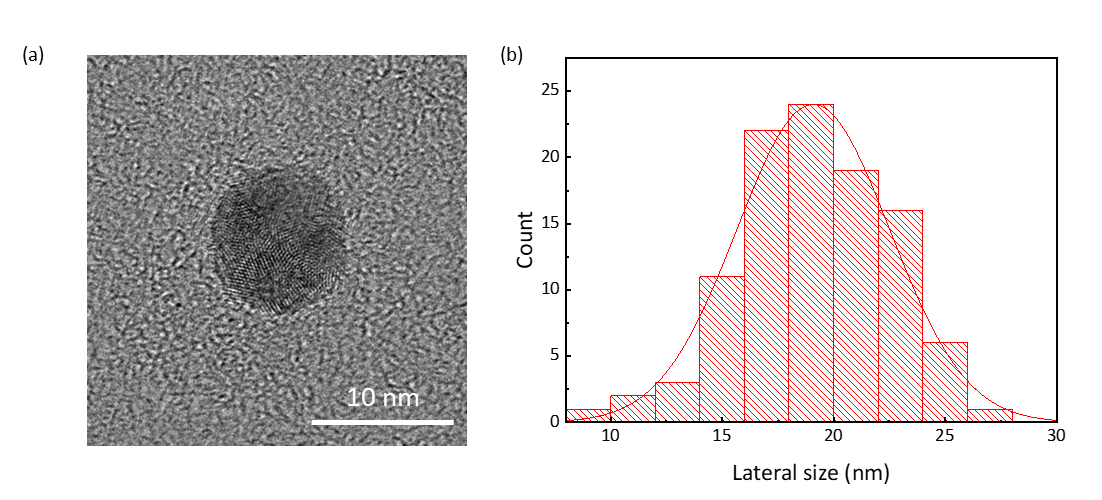


**Figure S2.** (a) TEM image and (b) size distribution of GQDs (100 GQDs measured).


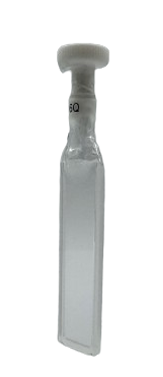


**Figure S3.** Photo image of quartz cuvette (L × W × H = 4.5 × 12.5 × 55 mm) with a 2 mm optical path length, 10 mm internal width, and a sample volume of 0.7 ml.


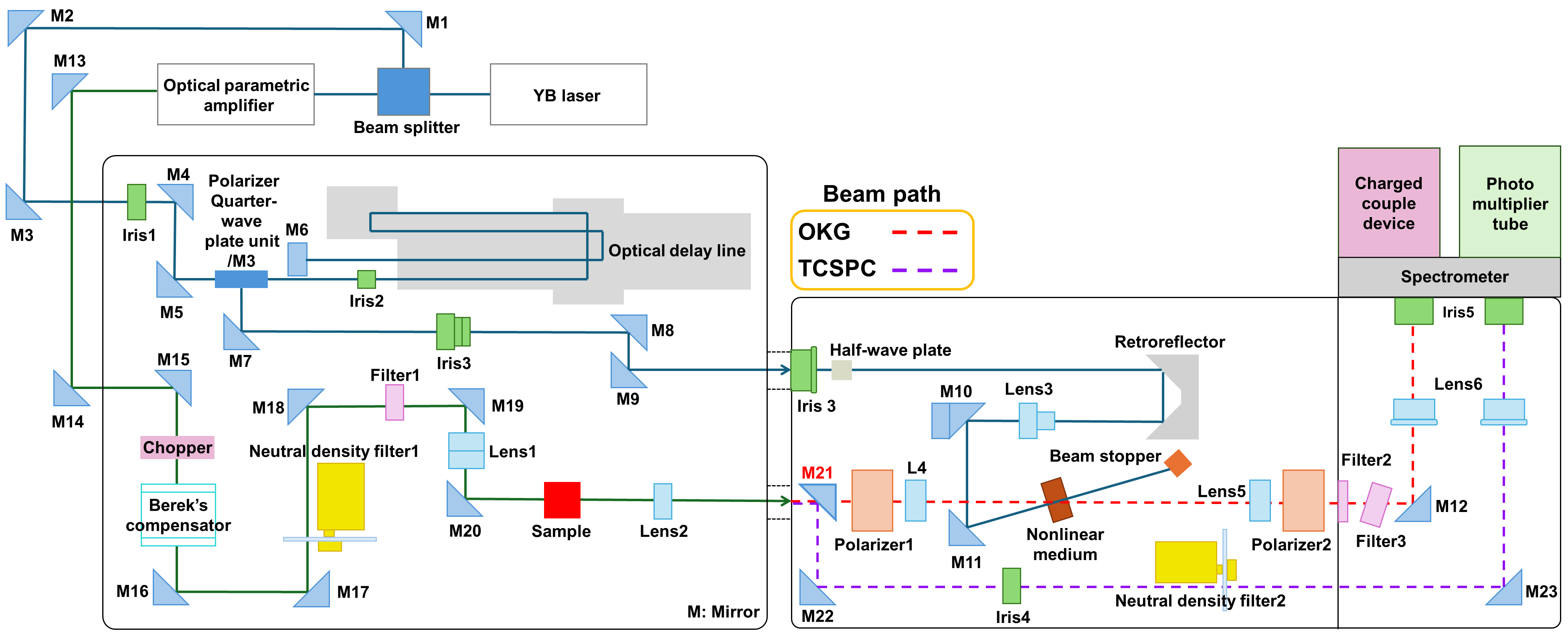


**Figure S4.** Optical layout of the transient fluorescence setups. The OKG beam path is depicted in red, while the TCSPC beam path is depicted in purple. The gated pulse is depicted in blue.

Fig. S4 illustrates the detailed optical and electronic configuration used for the combined TCSPC and OKG measurements. The excitation source is routed through an optical parametric amplifier to generate the desired wavelength. The emitted photoluminescence signal from the sample is collected and directed through the detection path. Beam splitters are used to divide optical paths, while beam stoppers are employed to block unwanted beams, with the notation explicitly distinguished to avoid ambiguity. By adjusting Mirror 21, the emission signal is selectively directed either to the TCSPC detection arm or to the OKG detection arm. For TCSPC measurements, the signal is detected by a photomultiplier tube and processed using commercially available TCSPC timing electronics, which record photon arrival times with picosecond temporal resolution. For OKG measurements, the gated emission is dispersed by a spectrometer and detected using a charge-coupled device. The relative delay between the pump and gate pulses is controlled by a motorized delay stage. This integrated configuration enables seamless switching between nanosecond- and sub-picosecond-resolved photoluminescence measurements within a single optical setup.


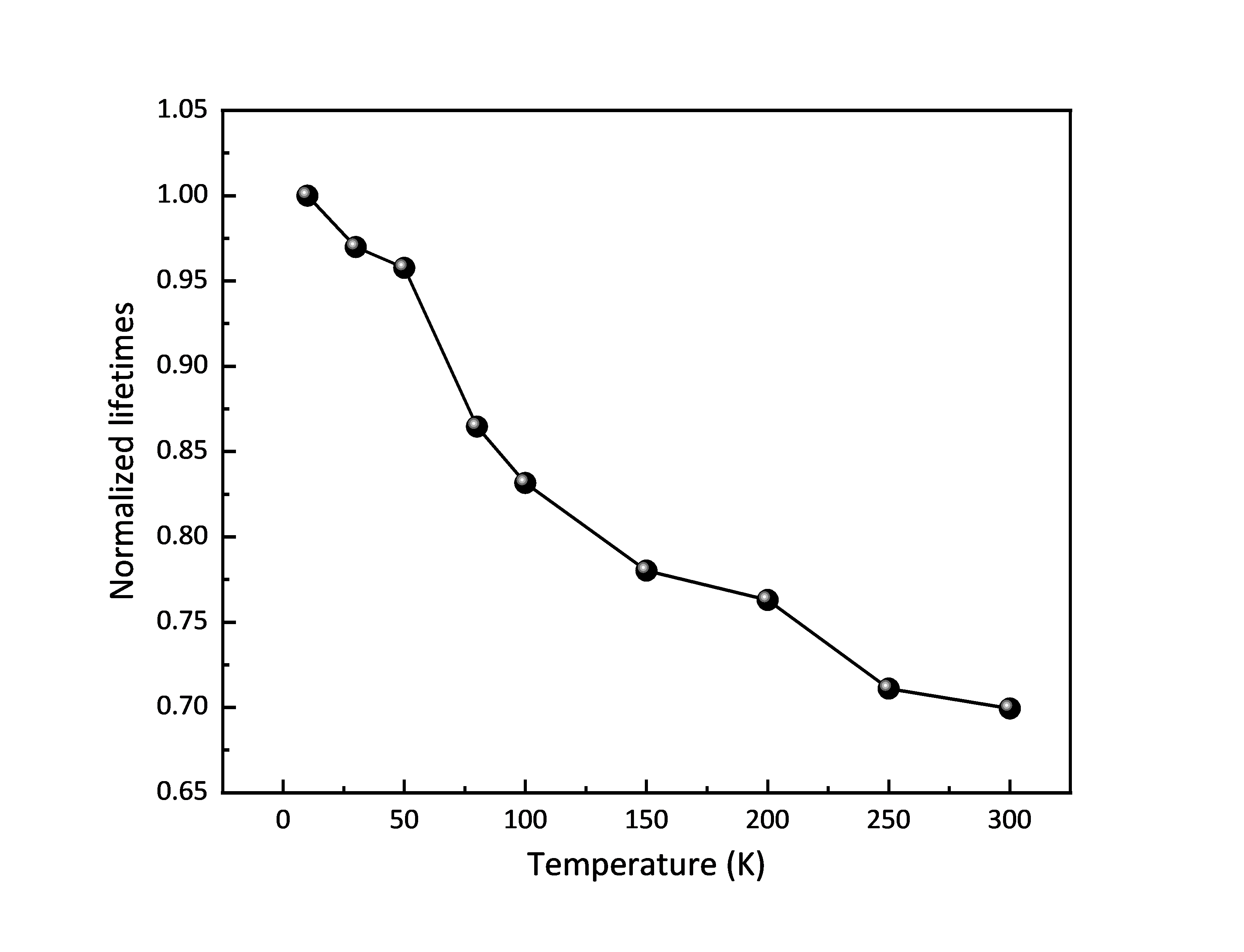


**Figure S5.** Normalized temperature-dependent TCSPC lifetimes decay curves of GQDs using drop-cast samples on Si substrates.

**Functional Group-Specific Ultrafast Dynamics from Optical Kerr-Gate Measurements**

Figs. S6–S9 present representative wavelength-dependent Kerr-gate decay profiles for selected emission bands, providing quantitative validation of the functional group-specific lifetime components discussed in the main text (Fig. 3). Each decay profile was fitted using mono- or dual-exponential functions to extract characteristic lifetimes associated with specific surface functional groups. The selected wavelengths correspond to the amine-related (549 nm), carboxyl/carbonyl-related (530 nm), hydroxyl/carbonyl-related (470 nm), and epoxy-related (550 nm) emission bands, respectively. The results confirm the presence of distinct ultrafast recombination dynamics for each functional group: amine exhibits a single ~21 ps decay; carboxyl/carbonyl displays dual components (~7/59 ps); hydroxyl/carbonyl shows dual components (~3/56 ps); and epoxy reveals a weak mono-exponential decay (~43 ps), consistent with its known non-radiative character. These data support the cascade relaxation model and trap-specific dynamics identified in the main Kerr-gate analysis.

**
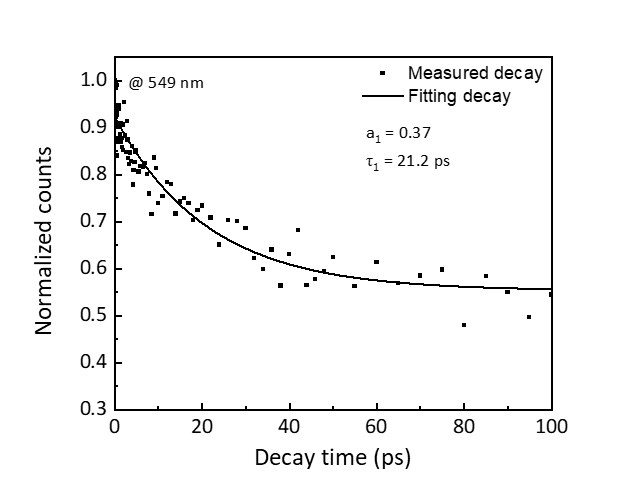
**

**Figure S6.** Decay profile at 549 nm fitted with a mono-exponential function (τ ≈ 21.2 ps).


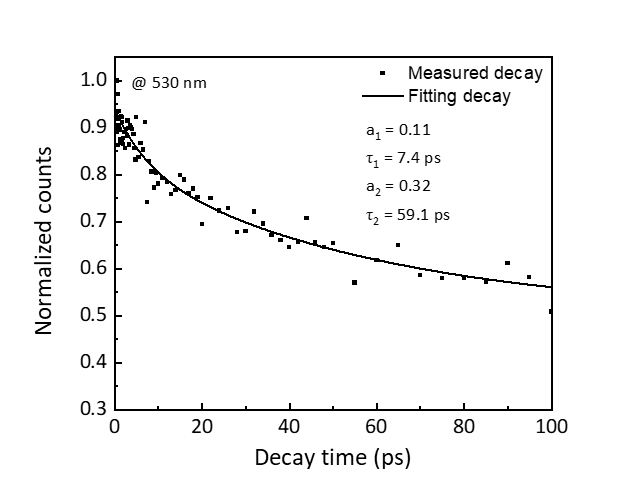

**Figure S7.** Decay profile at 530 nm fitted with a dual-exponential function (τ_1_ ≈ 7.4 ps and τ_2_ ≈ 59.1 ps).


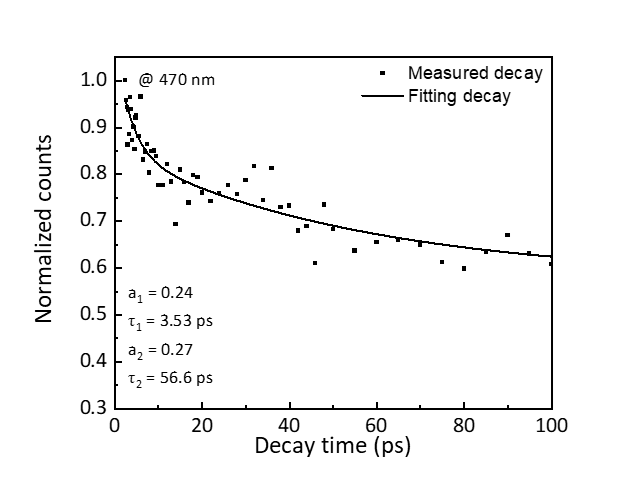


**Figure S8.** Decay profile at 470 nm fitted with a dual-exponential function (τ_1_ ≈ 3.53 ps and τ_2_ ≈ 56.6 ps).


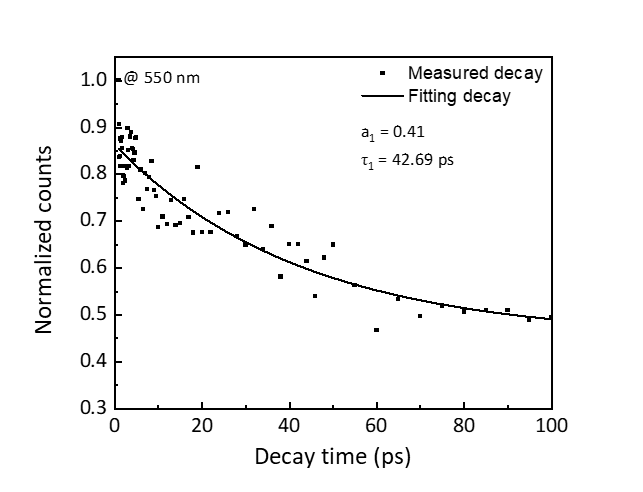


**Figure S9.** Decay profile at 550 nm fitted with a mono-exponential function (τ ≈ 42.69 ps).

**Temperature-Dependent Photoluminescence and Gaussian Deconvolution of GQDs**

Figs. S10 and S11 present the temperature-dependent PL spectra of GQDs (10–300 K) and their corresponding Gaussian deconvolution results. The raw spectra (Fig. S10) provide the full spectral evolution, revealing a progressive red-shift and broadening of Peak III with increasing temperature, while Peak I remains spectrally stable. The Gaussian-fitted spectra (Fig. S11) confirm that three distinct emission bands can be consistently resolved across all temperatures, supporting the multi-peak analysis applied in Fig. 4a. These results validate the assignment of temperature-dependent recombination behaviors for Peaks I and III discussed in the main text.


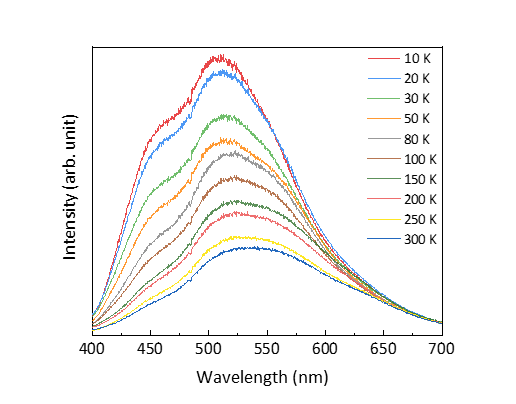


**Figure S10**. Cryogenic PL spectra of GQDs over the temperature range of 10–300 K.

**
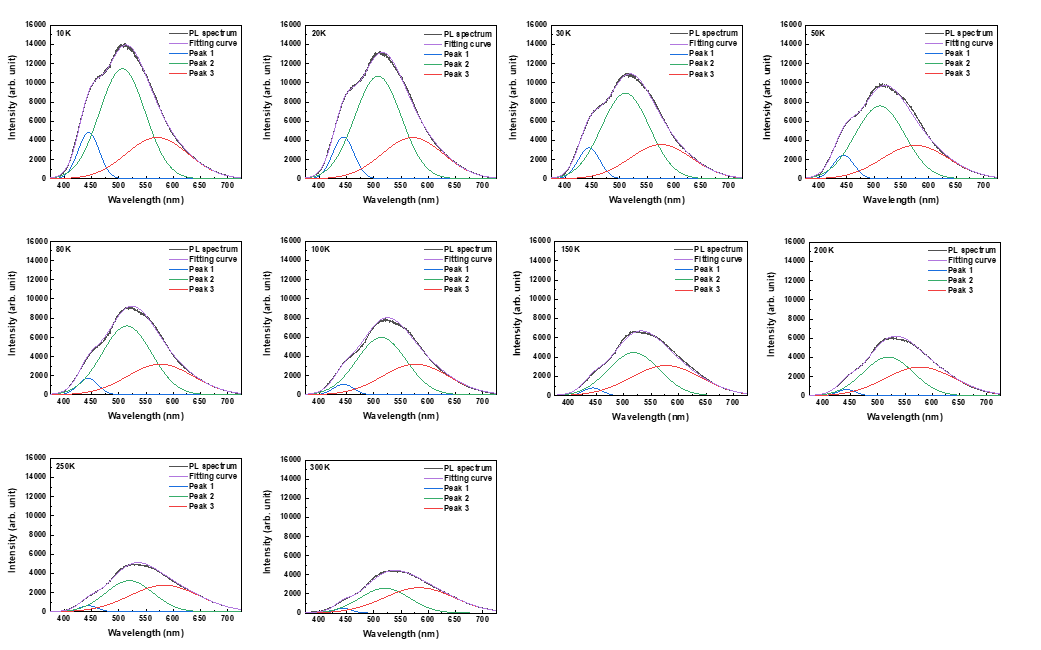
**

**Figure S11**. Gaussian-fitted PL spectra of GQDs at each temperature from 10 to 300 K.

**Power-Dependent Photoluminescence and Gaussian Deconvolution of GQDs at Room Temperature**

Figs. S12 and S13 present the power-dependent PL spectra of GQDs at room temperature and their corresponding Gaussian deconvolution results, supporting the α-factor analysis shown in Fig. 4d. The raw spectra (Fig. S12) demonstrate consistent spectral profiles across the excitation power range of 0.47–8 mW, enabling reliable Gaussian fitting. The fitted spectra (Fig. S13) confirm that the three characteristic emission bands are well-resolved at all powers, validating the extraction of integrated intensities for α-factor determination. These results ensure that the observed linear (Peak I) and super-linear (Peak III) power dependencies reflect intrinsic recombination behaviors rather than fitting artifacts or power-induced spectral shifts.


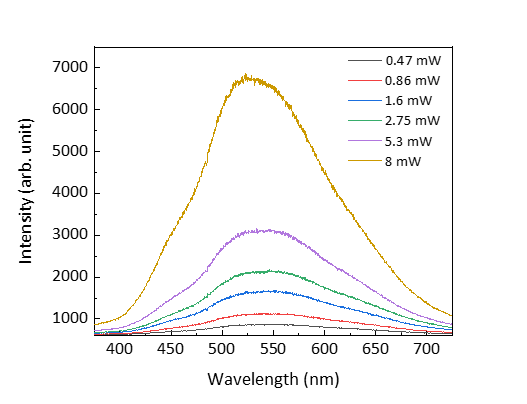


**Figure S12.** Room-temperature PL spectra of GQDs over the excitation power range of 0.47–8 mW.


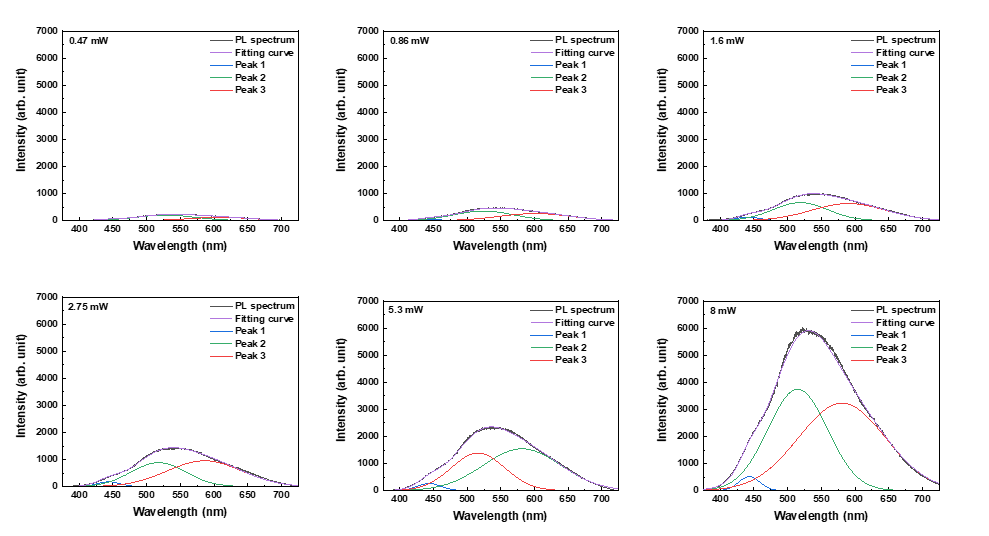


**Figure S13.** Gaussian-fitted PL spectra of GQDs at each excitation power from 0.47–8 mW.

**
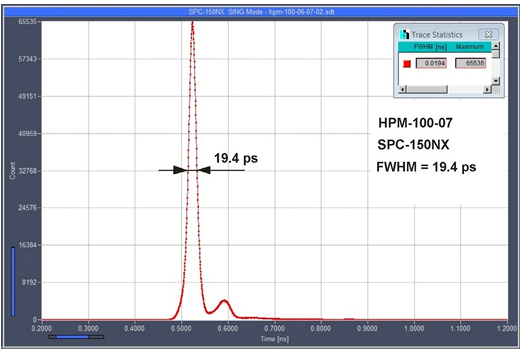
**

**Figure S14.** Instrument response function (IRF) of the TCSPC system measured 100-fs fiber laser.

**Table S1.** Specifications of Yb laser.

| **CARBIDE-CB3-40 W (Yb laser)** | |
| --- | --- |
| Center wavelength | 1030 ± 10 nm |
| Maximum output power | 40 W |
| Pulse duration | < 250 fs |
| Pulse duration tuning range | 250 fs – 10 ps |
| Beam diameter | 3.9 ± 0.4 mm |

**Table S2.** Specifications of time resolved fluorescence Kerr Gate and TCSPC module.

| **Kerr gate mode** | | **TCSPC mode** | |
| --- | --- | --- | --- |
| Spectral range | 250 – 1000 nm | Wavelength range (spectral range) | 220 – 820 nm |
| Delay range and resolution | 8 ns (8.3 fs) | Maximum measurement range | 5 μs |
| Temporal resolution | 400-500 fs | Temporal resolution | < 50 ps with high speed detector |
